# Bending Behaviour of Prestressed T-Shaped Concrete Beams Reinforced with FRP—Experimental and Analytical Investigations

**DOI:** 10.3390/ma15113843

**Published:** 2022-05-27

**Authors:** Mathias Hammerl, Benjamin Kromoser

**Affiliations:** Institute of Green Civil Engineering, University of Natural Resources and Life Sciences Vienna, Peter-Jordan-Straße 82, 1190 Vienna, Austria; benjamin.kromoser@boku.ac.at

**Keywords:** prestressed concrete, FRP, GFRP, UHPC, HPC, filigree structures, carbon concrete, pre-tensioned CFRP, resource efficiency, textile reinforcement, FRP anchorage

## Abstract

Materials such as high performance (HPC) or ultra-high performance concrete (UHPC), and fibre-reinforced polymer (FRP) reinforcement can be used to improve the resource efficiency in concrete construction by, for example, enabling the production of thin-walled structures. When building filigree concrete beams two essential factors must be considered: the low stiffness of the structure and the bond between the materials. By prestressing the structural stiffness is improved while an adequate concrete cover ensures sufficient bond strength. Based on this the bending behaviour of prestressed T-shaped beams reinforced with FRP, focussing on determining the influence of four parameters on the bearing capacity, bond behaviour and failure mode, is investigated in this paper. Comprehensive experimental investigations prove the potential of the approach and show that a reduction of the web thickness down to 40 mm, a lower concrete quality, and the use of glass FRP instead of carbon FRP allow a more resource-efficient structure while the applied prestressing leads to a higher utilisation of the high performance materials.

## 1. Introduction

The building industry has major impact at the environment and increasing efficiency and sustainability of the construction sector is therefore of highest relevance for further developments. With a global raw material consumption of about 50 Gt/year, the building industry is a major contributor to the climate problem [1]. Considering concrete construction in particular, 60% (approx. 30 Gt/year) of the raw material consumption can be attributed directly to this sector [1] with the cement industry itself responsible for about 8% of the global CO_2_ emissions [2]. Kromoser [3] and Reichenbach et al. [4] point out that the combination of high customisability and poor utilisation due to low costs is responsible for the inefficient use of material resources within the concrete industry. To increase the resource efficiency, two strategies are defined: (1) the optimisation of the used materials and (2) the optimisation of the structure itself, which can refer to the inner and outer structure [3,5]. A review on numerical and experimental investigations on topology optimisation can be found in [6]. Further information about sustainable design and construction with concrete can be found in [7].

The authors’ approach is to combine the two above mentioned strategies by using high-strength materials to build thin-walled concrete beams with an optimised cross section. The non-corrosive reinforcement made of carbon (CFRP) and glass fibre-reinforced polymer (GFRP) allows a reduction of the concrete cover while the high compressive strength of high (HPC) and ultra-high performance concrete (UHPC) leads to filigree structures due to optimised material utilisation. Stoiber et al. [8] conducted a life cycle assessment of CFRP reinforcement for concrete structures presenting the environmental competitiveness of CFRP through an exemplary comparison of three different bridges. Although the environmental impact of CFRP reinforcement per kg material is significantly higher than that of steel reinforcement, the carbon reinforced concrete bridge showed a lower global warming potential (GWP) due to better material utilisation and higher material properties compared to the steel reinforced concrete bridge and the bridge made of mild steel. Further considering the presumable longer life span of FRP-reinforced structures, the resource efficiency is even higher. A life cycle cost and environmental analysis of a bridge in Florida, conducted by Cadenazzi et al. [9], shows the ecological advantages of FRP compared to carbon steel reinforcement, mainly due to the reduction in maintenance costs. Stainless steel is presented to be the most competitive alternative to FRP with slightly higher costs.

When building lightweight structures with FRP reinforcement, two essential facts must be considered: the low bending resistance of filigree cross sections and the comparatively low stiffness yet high strength of the CFRP reinforcement resulting in high deflections of FRP reinforced structures. To counteract this behaviour, prestressing of the FRP reinforcement is essential to improve the bending resistance and increase the serviceability of these structures [10]. Design codes and guidelines published in recent years [11,12,13,14,15,16] support the implementation of prestressed CFRP in the construction industry, with numerous challenges needing to be considered especially when building thin-walled structures. Due to the influence of deviations on the load-bearing behaviour and the low concrete covers of often only 10 mm, a high accuracy must be achieved in the production process. Spalling of the concrete in areas of concentrated stresses as well as the load introduction of prestressed reinforcement in thin-walled structures poses an even greater challenge. Experimental investigations on slender cross sections reinforced with CFRP have shown that spalling of the concrete can lead to an early decrease of the stiffness of the structural elements [5,17], and cause early failure due to insufficient bond between the reinforcement and the concrete [10]. For this reason, extensive studies focussing on splitting failure when using textile reinforcement [18,19], and bond behaviour of CFRP bars were conducted in recent years. To complicate the matter, various surface treatments impede the investigations on the bond between FRP bars and concrete. In [20], the bond behaviour between subtractive milled CFRP bars and UHPC is presented, a broad study on tight-wrapped CFRP bars in four different concrete types can be found in [21], and Stark et al. [22] conducted a study on CFRP bars and cables in ultra-high performance fibre reinforced concrete (UHPFRC) with a special focus on prestressing and the transfer length in the load introduction area.

Although numerous investigations have been recently carried out on thin-walled structures reinforced with FRP [5,17,23,24,25,26], there are still numerous open questions especially about minimal dimensions while fully utilising the reinforcement. Therefore, further structured research regarding prestressed filigree FRP-reinforced structures is required. In [27], Terrasi presents several structural elements made of HPC reinforced with CFRP. It is pointed out, that for a rational use of CFRP, prestressing forces of about 65% of the tensile strength are required with preferably using CFRP bars or wires with smaller diameters of 3 to 6 mm (due to bond and load transfer issues). In [28,29], a focus is set on prestressed elements using mesh reinforcement. The investigations and applications on T-shaped, π-shaped and trapezoidal cross sections with thicknesses decreased down to 40 mm show the potential of these materials [28]. Doubly curved and folded plate sandwich panels using ultra-high performance fibre reinforced concrete (UHPFRC) and CFRP wires and bars are presented by Stark et al. [30]. Stresses up to 1350 MPa were applied within prestressing, resulting in a significant increase of the structural stiffness, underlining the positive effect of prestressing CFRP reinforced structures.

Following the introduction, the materials used in the presented investigations are described, giving detailed information on the used HPC and UHPC and focussing on the production, the components and the material properties. An overview of the CFRP bars and the mesh reinforcement made of GFRP and CFRP is presented. The final developed T-shaped cross section is based on the discussed preliminary investigations on the influence of different cross sections on the bearing behaviour and failure modes. The experimental investigations, the test configuration, the preparation of the beams including the prestressing process, the test setup and the results are described in Section 4. An analytical investigation based on the presented findings shows the accuracy and possibility of using a nonlinear analysis program for the calculation of reinforced concrete cross sections. A conclusion summarises the presented study.

## 2. Materials

In the presented investigations high performance materials were used. The advantageous material properties, such as the high compressive strength and stiffness of HPC and UHPC and the corrosion resistance and high tensile strength of FRP, are mandatory for the design of filigree structures with high load-bearing capacities. CFRP bars and textile CFRP and GFRP meshes were used as reinforcement.

### 2.1. Concrete

The constituents of the used HPC and UHPC are listed in Table 1. Fine-grained mixes with self-compacting characteristics were chosen to ensure adequate casting and a low air void content of the beams, although the “elephant skin” on the surface of the concrete impeded a proper deaeration [31]. Both concrete mixes were mixed in a conventional compulsory twin-shaft mixer at a prefabrication plant. While the HPC constituents were mixed all at once for about 180 s, the UHPC was mixed in two stages with the dry constituents first mixed for about 180 s before adding the wet constituents and mixing everything for another 300 s. For both mixes, the air content of the fresh concrete was determined to be around 1.1 Vol.-% [32], with the flow test [33] showing 780 and 760 mm for the HPC and UHPC, respectively. The water-binder ratio of the HPC had a value of 0.45 with a density of the fresh concrete of 2339 kg/m^3^, and 0.26 and 2393 kg/m^3^ for the UHPC, respectively. These densities differ slightly to the weights specified in Table 1 due to the entrapped air in the fresh concrete and possible inaccuracies in the weighing process of the raw materials at the prefabrication plant. In order to test the hardened concrete properties, as listed in Table 2, several additional specimens were cast and stored next to the beams to allow comparability. The concrete compressive strength f_cm,cube,45h_ was tested right before releasing the prestressing force about 45 h after casting while f_cm,cube_ and f_cm,cyl_ were tested 29 days post casting according to [34]. In addition, the flexural tensile strength ^#^ f_ctm,fl_ and the splitting tensile strength * f_ctm,sp_ were determined as stipulated in [35]. The axial tensile strength^+^ f_ctm_ was then calculated based f_ctm,fl_ and f_ctm,sp_ as described in [15] with the mean value of these calculations set as the axial tensile strength ^+^ f_ctm_ used for further investigations. Finally, the Young’s modulus was tested in accordance to [36]. Experimental tests on the fracture energy of the used mixtures were conducted and presented in [21].

### 2.2. Fibre-Reinforced Polymers

There is a wide variety of fibre-reinforced polymers used as embedded reinforcement in concrete structures. Reichenbach et al. [37] present a study, focussing on European manufacturers of one- and two-dimensional FRP reinforcement and their availability, composition and manufacturing. In general, carbon reinforcement shows the best mechanical properties with a tensile strength of up to 4000 MPa and a Young’s modulus of 230 GPa, whereas the reinforcement made of glass fibres is limited by a tensile strength of 1500 MPa and a significant lower Young’s modulus of 72 GPa. For this reason, prestressed, tight-wrapped CFRP bars (Rebar CCE8), produced by [38] were used as the main reinforcement in the presented investigations. Detailed information on the bond behaviour of the used bars based on pull-out-tests in four different concretes is presented in [21], where the bars used in this study are referred to as C2_D8. CFRP and GFRP meshes were further used as a secondary and shear reinforcement. Lower strength requirements for these structural elements allowed the use of reinforcement with a lower environmental impact. Within the manufactured specimens three different reinforcement meshes, produced by [38] were used. The CFRP meshes Q85/85-CCE21 and Q95/95-CCE38 as well as the GFRP mesh Q121/121-AAE38 differed in the reinforcement cross-sectional area per m, the axis distance of the single roving and the used fibre material. All reinforcements, both bars and meshes, were produced using epoxy resin as matrix material. A summary of the essential material properties is listed in Table 3.

## 3. Preliminary Investigations

**Figure 1:**
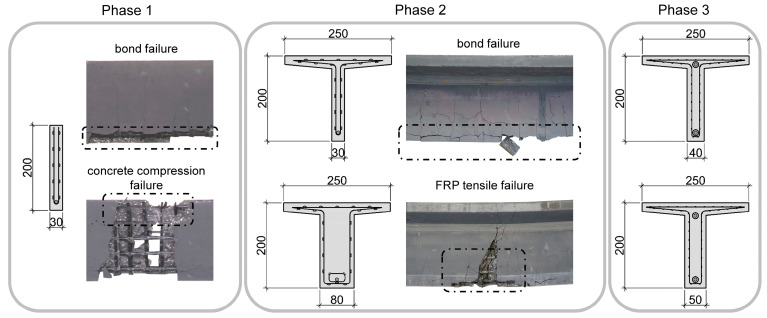
Overview of the preliminary investigations. Phase 1 showing rectangular beams with bond and concrete failure. Phase 2 showing bond and tensile failure of the FRP reinforcement, depending on the web thickness. Phase 3 representing the cross section of the current investigations.

In the preliminary investigations, various parameters were analysed to optimise the utilisation of the used high-performance materials. The extensive parameter study on rectangular concrete beams (Figure 1—Phase 1) is presented in [10]. Besides the concrete compressive strength, the prestressing force, the main reinforcement CFRP bar type and the end anchorage was varied. The investigations showed that a sufficient bond strength between CFRP bar and concrete is indispensable, as insufficient bond leads to early failure due to spalling of the concrete. Furthermore, a decrease in stiffness was determined after occurrence of the first cracks, resulting in higher mean crack widths. The beams with sufficient bond experienced failure due to crushing of the concrete in the compression area. The beams made of concrete with lower concrete compressive strength experienced bond failure and reached lower ultimate loads than the beams with the same reinforcement and UHPC. Pre-tensioning of the main CFRP reinforcement resulted in an improvement of the structural stiffness and serviceability. Analytical investigations of the rectangular beams with the interactive nonlinear cross-sectional analysis program INCA 2 [39] validated the experimental results. The calculated maximum bending moment amounted to 15 kNm, and was almost equal to the experimental failure moment, which was 14.9 kNm on average for the non-prestressed beams that failed due to crushing of the concrete [40].

Based on these findings the cross section was adapted resulting in a T-shaped beam (Figure 1—Phase 2) with two different web thicknesses to increase the compression zone and reach full utilisation defined by a rupture of the FRP reinforcement. The analytical investigations, presented in [40,41], show that the adapted geometry would result in a failure of the bottom roving of the mesh reinforcement. For the thicker beam, with a web thickness of 80 mm, the analytical results of 32.05 kNm agreed well with the experimental results lying between 27 and 37 kNm. The calculated bending moment of the thin-webbed beam was significantly lower compared to the experimental tests. This can be traced back to the fact that, although a sufficient bond strength of the used CFRP bars was determined in [21], the low concrete cover resulted in an early failure due to spalling of the concrete.

The described investigations show that a sufficient large compression zone and proper concrete cover can lead to a failure of the high-strength CFRP reinforcement. The final adaption of the concrete cross section therefore led to a web thickness of 50 mm, respectively 40 mm, see Figure 1—Phase 3. This dimension was chosen as adequate, since the beams with the thin webs in Phase 2 failed at approx. 80% of the maximum bending moment and an increase of the web thickness of 10 to 20 mm was considered sufficient to counteract the previously mentioned problems while simultaneously still ensuring a filigree structure.

## 4. Experimental Investigations

### 4.1. Testing Configurations

**Figure 2:**
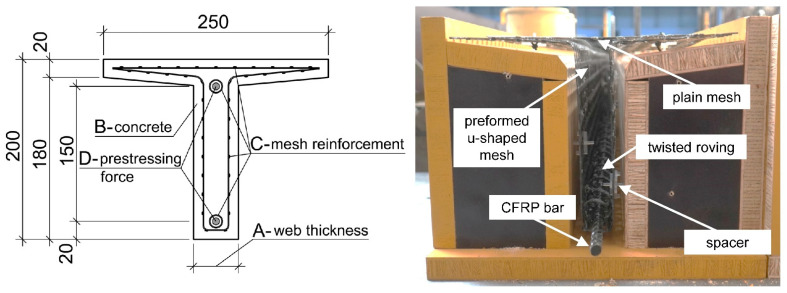
Top: Cross-section and varied parameters of the investigations; bottom: cross-sectional view of the reinforcement layout.

In this section, the configurations of the experimental investigations of T-shaped cross sections made of UHPC and HPC reinforced with CFRP and GFRP are presented. The basic cross section including the reinforcement layout and the parameters varied in the investigations can be seen in Figure 2. The web thickness (parameter A) of the T-shaped beams was chosen to be 50 and 40 mm and the concrete (parameter B) used was either an HPC or UHPC mixture. The secondary and shear reinforcement consisted of individually shaped GFRP and CFRP meshes, referenced to as parameter C. A U-shaped mesh reinforcement was used as shear reinforcement and a plain mesh was situated at the top of the beam as secondary reinforcement in the flange area. A twisted single flat roving was used as splitting tensile reinforcement and was loosely wrapped around the main reinforcement. All preformed FRP meshes were provided by the manufacturer and shaped during the production process. CFRP bars with a diameter of 8 mm were used as the main reinforcement. For the non-prestressed beams one bar was situated at the bottom of the beam in the tensile area. Within the prestressed beams two CFRP bars were used, one at the top and the other at the bottom of the beam, to assure an even prestressing and avoid cracking at the top of the beam. Due to the prestressing procedure both bars were prestressed equally. The prestressing force (parameter D) was varied between 30%, 50% and 70% of the characteristic tensile strength of the CFRP bar. An overview of the investigated beam configurations comprising in total 15 beams is listed in Table 4. The three parameters web thickness (A), concrete (B) and mesh reinforcement (C) were varied to investigate more resource-efficient alternatives, such as a thinner web thickness (concrete reduction), HPC instead of UHPC (cement ratio reduction) or a GFRP mesh instead of CFRP (environmental impact reduction).

### 4.2. Specimen Preparation and Prestressing

**Figure 3:**
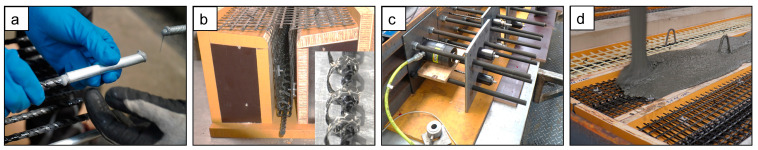
Specimen preparation and prestressing: (**a**) aluminium sleeve fixed to the CFRP bar using epoxy mortar; (**b**) reinforcement in the formwork and CFRP bar with twisted single yarn used as splitting tensile reinforcement; (**c**) prestressing process using hydraulic cylinder; (**d**) casting of the UHPC.

The beams were cast in a prefabrication plant. In a first step the CFRP bars were prepared for prestressing. Previous investigations showed that a mechanical sleeve wedge anchorage is suitable for introducing high prestressing forces into the CFRP bar. The anchorage method used for the investigations consisted of the CFRP bars, an aluminium sleeve with a length of 120 mm, an outer diameter of 12 mm and a thickness of 1 mm, epoxy mortar and a conventional steel wedge anchorage. The CFRP bars were cut to the desired length before the aluminium sleeves were placed at the ends and the cavities between CFRP bar and sleeve were filled with epoxy mortar, see Figure 3a. Further information on prestressing of FRP bars and the challenges involved can be found in [42,43,44].

Before the CFRP bars were mounted in the U-shaped FRP mesh, a twisted single roving, as shown in Figure 3b, serving as splitting tensile reinforcement was placed around them. Subsequently, the plain FRP mesh was fixed to the top of the U-shaped mesh using cable ties made of plastic. After applying the custom-made spacers [38], the reinforcement cage was placed in the previously carpentered formwork (Figure 3b) made of coated plywood. 

**Figure 4:**
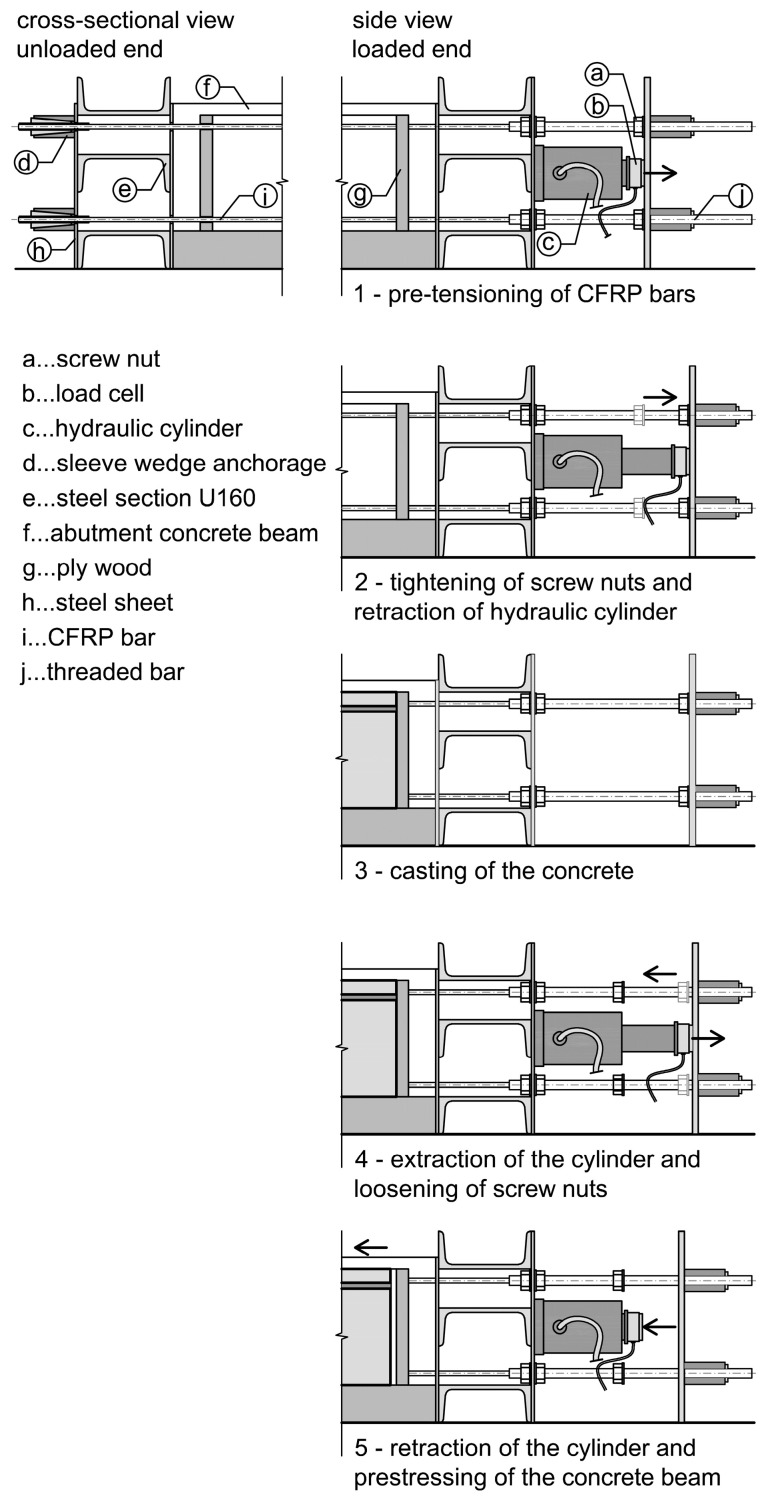
Prestressing process.

Reinforced concrete beams, steel profiles, and steel plates were used as abutments for pre-tensioning of the CFRP bars. The formwork was placed between the concrete beams and the steel sections were situated perpendicular at the end of the beams. Steel sheets with holes were used to fix the anchorage sleeves against the abutment system. At one end of the prestressing system, two steel sheets with four threaded bars were used as spacers (Figure 3c). To apply the prestressing force a hydraulic cylinder was placed between the steel sheets and the distance between the sheets was increased (Figure 4, step 1). The new position of the steel sheets was secured using screw nuts before the hydraulic cylinder was retracted and the prestressing force was induced into the abutment, see Figure 4, step 2. With this method, only simultaneous pre-tensioning of both CFRP bars was possible, see Figure 3c, with an equal distribution of the forces depending on the position accuracy of the hydraulic cylinder. To counteract prestress losses in the anchorage system all bars were overstressed with additional 2 kN. About 45 h after casting of the beams (Figure 3d and Figure 4, step 3) the pre-tensioning force was released onto the beams by extracting the hydraulic cylinder. First the cylinder was extracted and the CFRP bars were pre-tensioned to a point where the screw nuts could be loosened (Figure 4, step 4). Then the pressure of the cylinders was released within 20 to 30 s resulting in a full prestressing of the beams (Figure 4, step 5).

### 4.3. Test Setup

**Figure 5:**
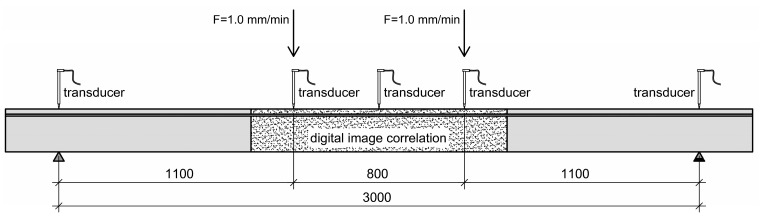
Test setup of the four-point bending tests (mm).

The test setup of the four-point bending tests can be seen in Figure 5. The span of the beams amounted to 3 m, with both supports rotatable. Layers of Teflon between the beam and the support reduced the friction between the two elements allowing unhindered sliding. The loads were applied in a displacement-controlled manner with a load rate of 1 mm/min at a distance of 1.1 m from the supports. Load cells integrated in the cylinder of the servohydraulic testing machine were used to measure the applied loads. The deflections were determined using five transducers situated at the supports, the load-introduction points, and in the middle of the beam. A non-contact photogrammetric measurement system with two cameras (DIC—digital image correlation) was used to monitor the strain and crack formation in the centre of the beam, where a constant bending moment was applied.

### 4.4. Results

The experimental results of the four-point bending tests will be discussed by comparing bending moment-deflection curves, the cracking behaviour and the failure modes. It must be mentioned that the own weight of the beams was not taken into account, since it was almost equal for all beams and neglectable causing a bending moment of only 0.40 kNm.

**Figure 6:**
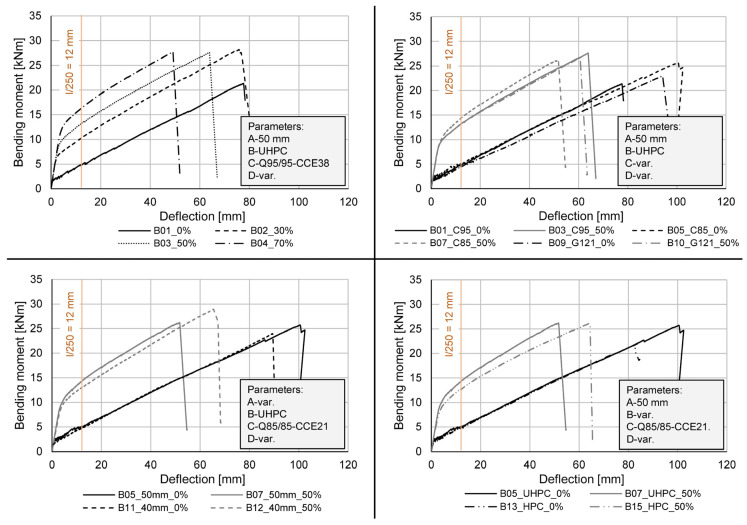
Comparison of bending moment-deflection curves based on the different parameters: (**top left**) prestressing force; (**top right**) mesh reinforcement and prestressing force; (**bottom left**) web thickness and prestressing force; (**bottom right**) concrete and prestressing force.

In Figure 6, bending-moment deflection curves are depicted, each presenting the influence of one or two investigated parameters. The beams with a web thickness of 50 mm, made of UHPC and reinforced with the carbon mesh reinforcement Q95/95-CCE 38 can be seen on the top left side. By applying a prestressing force on the beams, a clear improvement of the deflection behaviour is visible when comparing the bending moments at a deflection of 12 mm. Beam B01, for example, could only bear a bending moment of 5 kNm without prestressing, whereas the prestressed beams B02 and B04 reached bending moments of about 10 kNm and 16.5 kNm, respectively, at the same deflection. Due to the changed failure behaviour the ultimate bending moments of the prestressed beams were significantly higher than of those without prestressing. While beam B01 failed due to rupture of the bottom roving, the prestressed beams failed when the CFRP bar reached its ultimate tensile strength.

The diagram on the top right shows the influence of the different mesh reinforcements used as secondary and shear reinforcement in both non-prestressed and prestressed UHPC beams with a web thickness of 50 mm. As within the previous comparison a clear improvement of the deflection behaviour can be noticed for the prestressed beams, showing bending moments of 13 to 15 kNm compared to the 5 kNm of the non-prestressed variants at a deflection of 12 mm. The generally higher stiffness of beam B07 can be explained by a probably higher pre-tensioning force. Regarding the non-prestressed beams, a slightly lower stiffness of the beam reinforced with GFRP mesh reinforcement (B09) can be observed, whereas the ultimate bending moment shows no difference to that of the beam reinforced with the CFRP mesh Q95/95-CCE38. The highest achieved ultimate bending moment (beam B05) can be traced back to the higher tensile strength of the used CFRP mesh Q85/85-CCE21. It must be mentioned, that the test of beam B01 was stopped during the loading phase, due to overheating of the hydraulic aggregate and had to be restarted. An influence of the de- and reloading on the maximum bending moment cannot be excluded. For the prestressed beams no significant influence of the used mesh reinforcement is observed.

The bottom left diagram in Figure 6 presents the influence of the web thickness on the bearing behaviour of UHPC beams with a Q85/85-CCE21 mesh reinforcement. While the deflection behaviour of the non-prestressed beams does not show any differences, the beam with the higher web thickness (beam B05 compared to beam B11) reached a higher ultimate bending moment. The prestressed beam B12 shows a less stiff behaviour compared to B07, which can again be explained by the above-mentioned higher pre-tensioning force. The highest ultimate bending moment of all tests was reached by beam B12 (40 mm web thickness, UHPC, Q85/85-CCE21, 50% pre-tensioning force) with 28.9 kNm.

The impact of the different concrete compression strengths is depicted in the bottom right diagram of Figure 6. Even though the UHPC beams reached a higher ultimate bending load, no difference in stiffness can be observed for the non-prestressed beams. The prestressed HPC beam (B15) shows a flatter curve than the UHPC beam (B07) after a deflection of about 20 mm while having the same ultimate bending moment of around 26 kNm.

**Figure 7:**
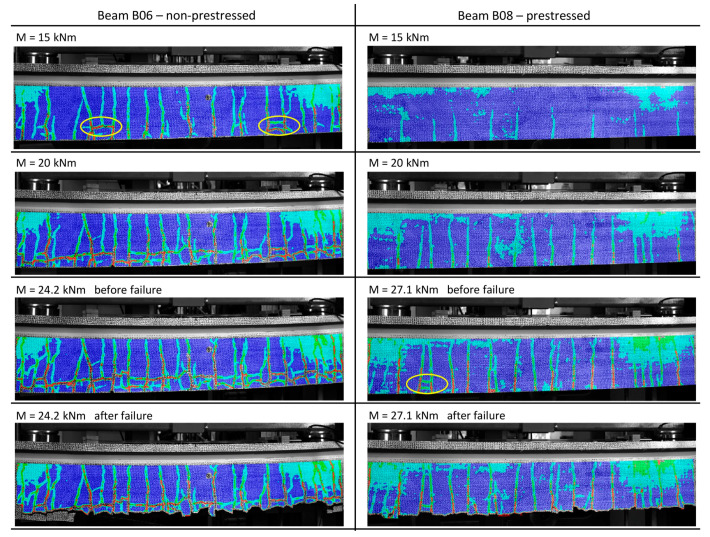
Crack patterns at different load-steps of a non-prestressed (B06) and prestressed beam (B08) with marking of first splitting cracks.

The cracking behaviour of the beams is discussed based on the occurrence of the first bending crack, the beginning of the first splitting cracks, the end of the cracking phase, and the bending crack width at a bending moment of 20 kNm. Using the DIC records all necessary information was obtained within postprocessing. The end of the cracking phase was determined by looking for a load step where no further bending cracks occurred in the detected area. Virtual extensometers were placed 10 mm above the bottom of the beam across each bending crack and the length change of each extensometer was measured for each load step. The cracking moment was defined to be the moment when the first crack reached a width of 0.01 mm. In addition to the bending cracks, the appearance of splitting cracks was also investigated, due to the high significance in terms of bond failure. In the presented investigations the splitting cracks were determined visually during the DIC postprocessing, and the corresponding bending moments, when they first occurred were listed. Cracking patterns of a non-prestressed (B06) and prestressed beam (B08) at different load steps can be seen in Figure 7. The positive influence of prestressing is clearly visible in the top pictures. While pronounced bending and splitting cracks are found on the non-prestressed beam, the prestressed beam shows only small initial cracks at a bending moment of 15 kNm. The subsequent increase of the bending moment leads to more splitting cracks within the non-prestressed beam. This is not the case for the prestressed variant, which is only characterised by an increase of the number of bending cracks. The crack patterns before failure show a strongly damaged non-prestressed beam and first splitting cracks in the prestressed beam. During failure the concrete cover at the bottom of both prestressed and non-prestressed beams was blasted off due to rupture of the bottom mesh roving or the CFRP bar.

**Figure 8:**
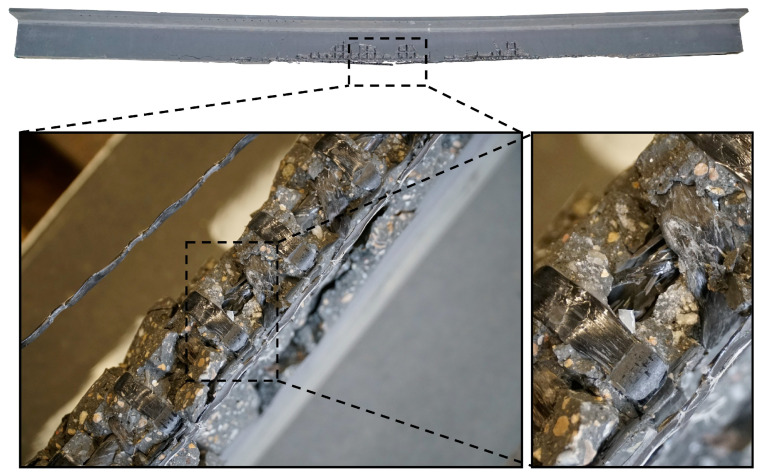
Failure mode of beam B3. Rupture of the CFRP bar.

An overview of the experimental tests and the results is given in Table 5. The ultimate bending moment M_u_ lies in a range between 21.3 to 28.9 kNm, with the prestressed beams generally showing higher M_u_-values due to the different failure mode, namely the rupture of the CFRP bar instead of the roving. The corresponding deflection at the ultimate bending moment D_M,u_ decreases with increasing prestressing. While the non-prestressed beams failed at a maximum deflection of 98.6 mm in the centre of the beam, the prestressed beam B08 showed a deflection of 48 mm at failure. As previously mentioned, the cracking behaviour was analysed by looking at and comparing the bending moments of the first bending crack occurrence M_be,cr_ and splitting crack occurrence M_sp,cr_ as well as the bending moment defining the end of crack formation M_cr,fin_ and the mean crack width at a bending moment of 20 kNm. For all configurations M_be,cr_ increased stepwise with the applied prestressing force. When looking at the beams with a prestressing level of 70%, for example, the tests showed a comparable M_be,cr_ of 9.8 kNm and 10.4 kNm. For the non-prestressed HPC beams and the beams with a web thickness of 40 mm, the splitting crack occurred earlier compared to the other configurations, however prestressing resulted in a significant increase of the M_sp,cr_ for these beams. In general, the beams reinforced with the carbon mesh with the larger roving axis distance, showed earlier splitting cracks compared to the other beams. The crack initiation phase ended at lower bending moments M_cr,fin_ for the non-prestressed beams. The highest M_cr,fin_ was observed in the specimens with the mesh reinforcement G121 made of alkali-resistant glass (AR-glass). The assessment of the crack widths at a bending moment of 20 kNm (w20_kNm_, Table 5 second-last column) shows the positive influence of prestressing, reducing the crack widths significantly. For the non-prestressed beams, the configuration with the small-meshed carbon textile showed the smallest crack widths, while the beams with HPC, GFRP mesh and the large-meshed CFRP textile showed clear inferior cracking behaviour. The influence of the prestressing diminished the differences within the cracking behaviour of the specimens with the various investigated parameters, with all specimens prestressed to a level of 50% showing crack widths between 0.37 to 0.27 mm, with the exception of beam B15, made of HPC with a mean crack width of 0.44 mm. The two different failure modes which occurred were either rupture of the bottom roving (non-prestressed beams) or rupture of the CFRP bar (prestressed beams). In the case of the beams reinforced with the Q121/121-AAE38 mesh, the non-prestressed beam also failed due to rupture of the CFRP bar. The failure mode CFRP rupture is depicted for beam B3 in Figure 8.

## 5. Analytical Investigations

**Figure 9:**
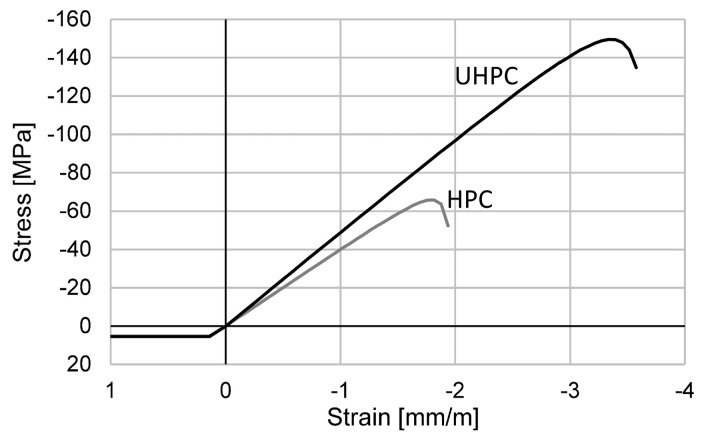
Stress–strain behaviour of the HPC and UHPC.

**Figure 10:**
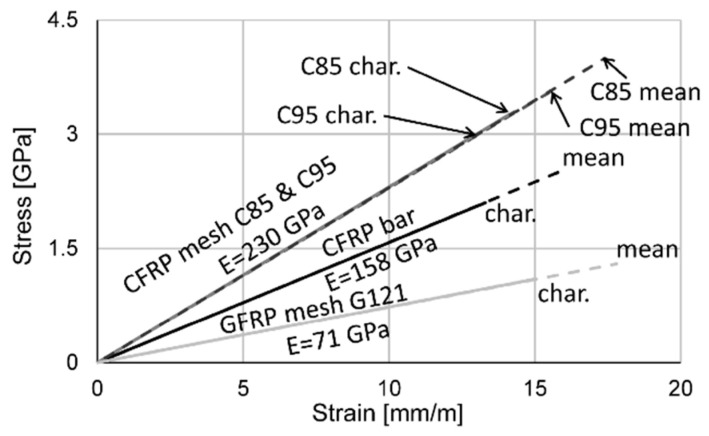
Stress–strain behaviour of the FRP reinforcement (Linear).

The analytical investigations were conducted using the nonlinear cross-sectional analysis program INCA 2 [39], using the following procedure:

1. Definition of the geometry of the different configurations using points for the centre of the reinforcement, and points and lines for the cross section.
2. Implementation of the material behaviour according to the experimental investigations and the manufacturer specifications, as listed in Table 2 and Table 3. The used stress–strain behaviour dependence is visualised in Figure 9 and Figure 10.
3. Recalculation of every beam, further referred to as beam B01_char and B01_mean using the characteristic or mean material properties of the reinforcement (Table 3) for the nonlinear analysis.
4. Loading the cross section with the pre-tensioning force with an initial strain in the CFRP bars. Based on the characteristic breaking strain of 13.3 mm/m, the strain for prestressing was calculated with 3.99 mm/m (30%), 6.65 mm/m (50%) and 9.31 mm/m (70%).

**Figure 11:**
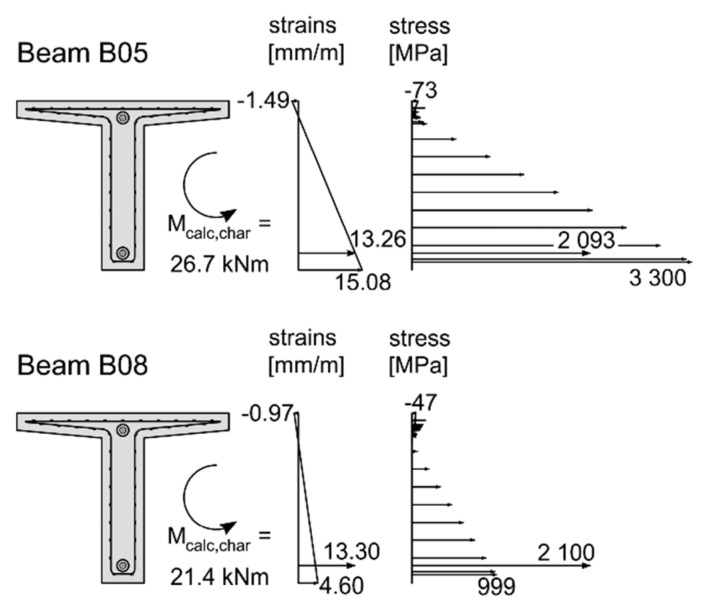
Stress and strain distributions of beams B05 and B08, calculated using characteristic material properties of the reinforcement.

The results of the analytical investigations are summarised in Table 6. For all configurations two calculations based on the characteristic (calc_char._) and the mean (calc_mean_) reinforcement properties were conducted. Exemplary stress and strain distributions of beam B05 and beam B08, calculated using the characteristic material properties (calc_char._) of the reinforcement are presented in Figure 11. In beam B05, the maximum characteristic tensile stress (3300 MPa) was reached in the bottom roving (rupture in the experiment) of the mesh reinforcement with a corresponding stress in the CFRP bar of 2093 MPa, lying slightly below the characteristic tensile strength of 2100 MPa. The prestressed beam B08 reached the maximum calculated bending moment (calc_char._) of 21.4 kNm right before failure of the CFRP bar at 2100 MPa. Considering the ultimate bending moment M_u_ with the accompanying tensile stresses in the bottom roving σ_roving_, in the CFRP bar σ_bar_ and the compressive stress in the concrete σ_concrete_, as well as the cracking moment M_cr_ a comparison of the experimental and analytical investigations is possible. For the prestressed beams with the mesh reinforcement Q95/95-CCE38 the ultimate bending moments M_u_ of the experimental tests correspond very well with the calculated results using the characteristic and mean values, whereas the results for the non-prestressed beam B01 differ significantly. This can be, with a high probability, traced back to the above-mentioned problems during the loading phase and not the fact that the beam was not prestressed. When considering the other non-prestressed beams (B05, B06, B09 and B11) the difference between calculated and experimental (characteristic values) results is low, except for beam B13. Regarding the prestressed beams, the calculated ultimate bending moments agree well with the experimental results. Most of the results and the corresponding stresses confirm the failure modes obtained in the experimental tests: rupture of the roving and rupture of the CFRP bar. In the case of beams B11 and B13, the analytical results when using the mean values (CFRP bar and concrete compression failure, respectfully) do not confirm the experimental failure modes (failure of the mesh rovings).

## 6. Conclusions

The presented research shows an extensive experimental study on T-shaped beams made of HPC and UHPC with prestressed CFRP bars and reinforced with FRP meshes investigating the load-bearing behaviour in four-point bending tests. The analytical study using a nonlinear cross-sectional analysis program confirms that a calculation of these structures is possible yet needs in-depth knowledge of the material properties to ensure proper results. Even though the construction and standard-conform implementation of filigree structures comes with a challenge, especially in regard to bond, the presented results show the optimisation potential and possible analytical prediction of the failure behaviour. The feasibility and possible increase of the structural stiffness by prestressing thin-walled FRP reinforced beams with a web thickness of only 40 to 50 mm is confirmed and the influence of the parameters investigated in the tests can be summarised as follows:

- A reduction of the web thickness to a minimum (parameter A) was presented, ensuring failure of the reinforcement without significant bond problems. The ultimate bending moment M_u_ of the UHPC beams reinforced with a CFRP bar of 8 mm and the textile reinforcement Q85/85-CCE21 with a web thickness of 40 mm is comparable to the beams with a web thickness of 50 mm. The prestressed beam with this configuration even showed the highest M_u_ of all tests. Regarding the cracking behaviour a negative influence of the small web thicknesses is observed, presenting itself through an earlier occurrence of splitting cracks.
- The prestressed beams with different concrete compressive strengths (parameter B) show equal M_u_, with the bending stiffness of the HPC beams inferior to that of the UHPC beams. For the non-prestressed beams an earlier failure of the HPC beam was observed. The easier workability and lower environmental impact however still justify the use of HPC in filigree structures, with the above-mentioned disadvantages counteracted by optimised prestress levels.
- The variation of the mesh reinforcement (material, roving axis distance and roving cross-sectional area) shows no significant differences in the bearing behaviour, especially for the prestressed beams. The non-prestressed beams reinforced with a GFRP mesh show a lower bending stiffness (4 mm higher deflection at a bending moment of 15 kNm, see Figure 6) compared to the beams reinforced with CFRP meshes. Regarding the cracking behaviour the CFRP meshes with small roving axis distance (Q85/85-CCE21) are preferable, due to higher moments when the first bending and splitting cracks occur.
- For all configurations the prestressing of the CFRP bar shows an improvement of the load-bearing behaviour. The ultimate bending moment M_u_ is increased due to full utilisation of the CFRP bar. In addition, a significant improvement of the structural stiffness and the cracking behaviour in general is observed.

Based on these findings it can be said that prestressing of thin-walled structures increases the utilisation of the used materials. The bearing mechanism can be controlled and depending on the cross section a failure of the concrete compressive zone, rupture of the FRP mesh or failure of the CFRP bar can be achieved. Nevertheless, a sufficient bond behaviour is necessary to fully exploit the reinforcement material. The investigations show that the resource efficiency can be improved by further optimisation of the cross section, proper utilisation of the concrete and use of alternative mesh reinforcements made from GFRP with lower environmental impact. Another possible alternative would be the use of GFRP as the prestressing bar, even though the long-term strength of these material should be considered, since it is inferior to CFRP. All these optimisations can be based on an analytical investigation using a nonlinear analysis program. In addition to the bending capacity the resistance against shear forces, cyclic loading, fatigue is also essential for the assessment of the potential of thin-walled structures. The simultaneously conducted extensive investigations on the shear strength will be discussed in future works in combination with shear strength investigations of deck slab elements. The presented results show that the failure modes and bending capacity can be predicted. Based on the knowledge achieved in these investigations full-scale bending tests on two slab elements with a length of 8.4 m, a width of 1 m and a height of 0.2 m will be conducted using GFRP and a CFRP meshes as shear and secondary reinforcement.

## Figures and Tables

**Table 1:** Constituents of the used concrete mixtures in kg/m^3^.

Ingredients	HPC [kg/m^3^]	UHPC [kg/m^3^]
Water	163	170
Superplasticiser	5	32
Defoamer	1	1
Cement	420	712
Microsilica	-	143
Limestone powder	100	285
Quartz sand 0–4 mm	1640	1030

**Table 2:** Properties of the hardened HPC and UHPC in MPa.

Properties (Abbreviations)	Specimen Size [mm]	HPC [MPa]	UHPC [MPa]
f_ctm,fl_^#^	160 × 40 × 40	11.4 ^#^	10.6 ^#^
f_ctm,sp_ *	Ø100 × 200	5.3 *	6.3 *
f_ctm_^+^	-	5.1 ^#^/5.3 */**5.2** ^+^	4.7 ^#^/6.3 */**5.5** ^+^
E_cm_	Ø150 × 300	36,654	44,506
f_cm,cube,45h_	100 × 100 × 100	47.3	92.0
f_cm,cube_	150 × 150 × 150	65.9	149.6
f_cm,cyl_	Ø150 × 300	60.3	162.3

**Table 3:** Material properties of the used FRP reinforcement according to the manufacturer [38].

Properties	Rebar CCE 8	Q85/85-CCE21	Q95/95-CCE38	Q121/121-AAE38
Dimension	1D	2D	2D	2D
Fibre/Matrix material	Carbon/Epoxy resin	Carbon/Epoxy resin	Carbon/Epoxy resin	AR-glass/Epoxy resin
Roving axis distance	-	21 mm	38 mm	38 mm
Cross section of the roving/planar reinforcement	50.2 mm^2^	1.81 mm^2^/85 mm^2^/m	3.62 mm^2^/95 mm^2^/m	4.62 mm^2^/121 mm^2^/m
Mean tensile strength	2500 MPa	4000 MPa	3600 MPa	1300 MPa
Char. tensile strength	2100 MPa	3300 MPa	3000 MPa	1100 MPa
Mean Young’s modulus	158 GPa	230 GPa	230 GPa	73 GPa
Mean elongation at break	15.8‰	17.4‰	15.7‰	17.8‰
Char. elongation at break	13.3‰	14.3‰	13.0‰	15.1‰

**Table 4:** Overview of testing configurations.

Parameter		Beam No.														
		1	2	3	4	5	6	7	8	9	10	11	12	13	14	15
A—web thickness	50 mm															
	40 mm															
B—concrete	UHPC															
	HPC															
C—mesh reinforcement	Q95/95-CCE38															
	Q85/85-CCE21															
	Q121/121-AAE38															
D—prestressing force	0 kN = 0 MPa = 0%															
	31.7 kN/630 MPa/30%															
	52.8 kN/1050 MPa/50%															
	73.9 kN/1470 MPa/70%															

**Table 5:** Overview of experimental test results and failure modes: ultimate bending moment M_u_; deflection at ultimate bending moment D_M,u_; cracking moment M_cr_; splitting moment M_split_; bending moment at the end of the cracking initiation phase M_cr,fin_. Highest and lowest values are highlighted in bold.

Beam No.	M_u_	D_M,u_	M_be,cr_	M_sp,cr_	M_cr,fin_	W_20kNm_	Failure Mode
	[kNm]	[mm]	[kNm]	[kNm]	[kNm]	[mm]	[-]
B01_UHPC_C95_50_0%	**21.3**	76.6	1.7	10.1	8.2	**0.70**	Roving
B02_UHPC_C95_50_30%	28.2	75.6	5.4	18.2	14.5	0.41	Bar
B03_UHPC_C95_50_50%	27.6	64.1	7.1	15.5	14.5	0.37	Bar
B04_UHPC_C95_50_70%	27.7	49.4	9.8	19.5	15.3	**0.20**	Bar
B05_UHPC_C85_50_0%	25.7	98.4	2.5	9.7	10.3	0.40	Roving
B06_UHPC_C85_50_0%	24.2	**98.6**	1.7	12.6	8.3	0.53	Roving
B07_UHPC_C85_50_50%	26.2	51.6	8.6	23.9	19.3	0.34	Bar
B08_UHPC_C85_50_70%	27.1	**48.0**	**10.4**	**25.1**	19.5	0.23	Bar
B09_UHPC_G121_50_0%	22.9	94.4	**1.5**	10.0	18.4	0.57	Bar
B10_UHPC_G121_50_50%	26.6	60.7	7.7	22.2	**20.6**	0.32	Bar
B11_UHPC_C85_40_0%	24.0	87.6	1.6	8.1	**8.0**	0.43	Roving
B12_UHPC_C85_40_50%	**28.9**	65.5	7.9	16.3	13.7	0.27	Bar
B13_HPC_C85_50_0%	22.0	82.9	**1.5**	**7.5**	9.3	0.62	Roving
B14_HPC_C85_50_30%	27.6	80.8	4.9	18.8	14.5	0.46	Bar
B15_HPC_C85_50_50%	26.1	65.0	7.8	21.0	15.8	0.44	Bar

**Table 6:** Results of the analytical investigations (calculated failure is highlighted in bold, experimental failure is written in italic).

	M_u_			M_cr_		σ_roving_		σ_bar_		σ_concrete_	
	[kNm]			[kNm]		[MPa]		[MPa]		[MPa]	
	exp	calc_char._	calc_mean_	exp	calc	calc_char._	calc_mean_	calc_char._	calc_mean_	calc_char._	calc_mean_
B01_UHPC_C95_50_0%	21.3	27.2	32.9	1.7	2.1	***3000***	***3600***	1979	2394	−71	−85
B02_UHPC_C95_50_30%	28.2	26.3	31.7	5.4	6.6	2230	2810	***2100***	***2500***	−66	−80
B03_UHPC_C95_50_50%	27.6	24.3	29.8	7.1	9.9	1593	2178	***2100***	***2500***	−57	−72
B04_UHPC_C95_50_70%	27.7	22.3	27.9	9.8	13.2	956	1550	***2100***	***2500***	−47	−63
B05_UHPC_C85_50_0%	25.7	26.7	31.8	2.5	2.0	***3300***	*3911*	2093	**2500**	−73	−86
B06_UHPC_C85_50_0%	24.2	26.7	31.8	1.7	2.0	***3300***	*3911*	2093	**2500**	−73	−86
B07_UHPC_C85_50_50%	26.2	23.0	28.1	8.6	9.6	1655	2267	***2100***	***2500***	−56	−71
B08_UHPC_C85_50_70%	27.1	21.4	26.5	10.4	12.7	999	1614	***2100***	***2500***	−47	−62
B09_UHPC_G121_50_0%	22.9	22.8	27.1	1.5	2.0	1011	1196	***2100***	***2500***	−66	−78
B10_UHPC_G121_50_50%	26.6	21.4	25.8	7.7	9.8	506	694	***2100***	***2500***	−54	−68
B11_UHPC_C85_40_0%	24.0	26.8	31.9	1.6	1.7	*3262*	*3861*	**2100**	**2500**	−72	−86
B12_UHPC_C85_40_50%	28.9	23.3	28.4	7.9	9.9	1637	2239	***2100***	***2500***	−56	−70
B13_HPC_C85_50_0%	22.0	26.7	29,3	1.5	1.7	*3267*	*3574*	**2100**	2310	−63	**−66**
B14_HPC_C85_50_30%	27.6	24.7	29.7	4.9	6.4	2291	2895	***2100***	***2500***	−57	**−66**
B15_HPC_C85_50_50%	26.1	23.1	28.2	7.8	9.6	1635	2243	***2100***	***2500***	−51	−62

## Data Availability

All data contained with the article.
